# Ionic Diffusion‐Driven Ionovoltaic Transducer for Probing Ion‐Molecular Interactions at Solid–Liquid Interface

**DOI:** 10.1002/advs.202103038

**Published:** 2021-10-31

**Authors:** Junghyup Han, Sun Geun Yoon, Won Hyung Lee, Huding Jin, Yong Hyun Cho, Youn Sang Kim

**Affiliations:** ^1^ School of Chemical and Biological Engineering and Institute of Chemical Processes College of Engineering Seoul National University Gwanak‐gu Seoul 08826 Republic of Korea; ^2^ Program in Nano Science and Technology Graduate School of Convergence Science and Technology Seoul National University Gwanak‐gu Seoul 08826 Republic of Korea; ^3^ Advanced Institute of Convergence Technology Suwon 16229 Republic of Korea

**Keywords:** electrolyte–semiconductor interfaces, interfacial potential, ion–charge carrier interaction, ion‐ligand complexation, ion‐specific adsorption

## Abstract

Ion–solid surface interactions are one of the fundamental principles in liquid‐interfacing devices ranging from various electrochemical systems to electrolyte‐driven energy conversion devices. The interplays between these two phases, especially containing charge carriers in the solid layer, work as a pivotal role in the operation of these devices, but corresponding details of those effects remain as unrevealed issues in academic fields. Herein, an ion–charge carrier interaction at an electrolyte–semiconductor interface is interrogated with an ion‐dynamics‐induced (ionovoltaic) energy transducer, controlled by interfacial self‐assembled molecules. An electricity generating mechanism from interfacial ionic diffusion is elucidated in terms of the ion–charge carrier interaction, originated from a dipole potential effect of the self‐assembled molecular layer (SAM). In addition, this effect is found to be modulated via chemical functionalization of the interfacial molecular layer and transition metal ion complexation therein. With the aiding of surface analytic techniques and a liquid‐interfacing Hall measurement, electrical behaviors of the device depending on the magnitude of the ion‐ligand complexation are interrogated, thereby demonstrating the ion–charge carrier interplays spanning at electrolyte–SAM‐semiconductor interface. Hence, this system can be applied to study molecular interactions, including chemical and physical influences, occurring at the solid–liquid interfacial region.

## Graphical abstract

Ionic diffusion‐induced ionovoltaic transducer (IIT) is an ideal tool for interrogating interfacial molecular layer effect at the electrolyte–semiconductor interface. Complexation of ferric ion and catechol group is detected by electrical output changes and verified by surface analytic techniques and liquid‐interfacing Hall measurement. IIT shows promising potential as a tool for probing interfacial phenomena and applicability as novel ion sensors.

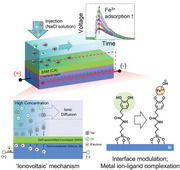

## 1 Introduction

Ion–surface interactions at liquid–solid interfacial boundary are one of the key principles for operating liquid‐interfacing devices ranging from electrochemical devices (e.g., battery, electrochemical capacitor) to electronic devices (e.g., bio‐/chemical FET sensors and electrolyte‐gated synaptic transistors).^[^ ^1^, ^2^ ^]^ Recently, these interactions also played an essential role in water motion‐driven electricity‐generating phenomena.^[^ ^3^, ^4^ ^]^ As a liquid electrolyte was adjoined with a solid electrode, liquid dynamics‐induced collective ionic motions near the interface can trigger unusual electrostatic potential effect in solid electrode, thereby inducing electric current generation in an electrical circuit.^[^ ^5^, ^6^, ^7^, ^8^, ^9^, ^10^ ^]^ Detailed origins and processes of their ion–surface interactions, which bring about the current flows, are attracting much attention in academic fields.^[^ ^4^, ^11^ ^]^ Among those effects, an interfacial ion dynamics‐driven electricity‐generating effect, called an ionovoltaic generation, was identified on an electrolyte/dielectric (self‐assembled molecular layer (SAM) and insulating layer)/electrode structure.^[^ ^9^, ^12^ ^]^ This concept was demonstrated in branches of various liquid motions, such as dynamic droplet motions, water evaporation, and capillarity infiltration.^[^ ^7^, ^9^, ^13^, ^14^, ^15^, ^16^, ^17^, ^18^, ^19^ ^]^ Specifically, an interfacial dipole effect from the orderly deposited molecular layer was proved as a foundation of ion–charge carrier interactions for the electricity generation in terms of the driving mechanism.^[^ ^9^, ^12^ ^]^ Moreover, it was verified by computational simulation results that the intrinsic dipole potential emanating from SAM enables ions adsorbing on SAM to drive simultaneous charging of compensating charges in electrolyte–SAM (insulator)‐semiconductor (ESS) interface. Lately, this was experimentally proved through a liquid‐adjoining Hall measurement in ESS structure, which verified that the ion adsorption on SAM could offer a self‐gating (without an external gate potential) effect, thereby accumulating charge carriers at the SAM–semiconductor interface.^[^ ^2^, ^20^ ^]^ These results suggested that, as the semiconductor has a less charge screening capability than a metallic electrode, it can sensitively vary its charge carrier density near the SAM–semiconductor interface depending on the electrolyte condition.^[^ ^2^, ^21^ ^]^ Hence, in the same line with various water‐motion‐induced electricity‐generating phenomena, ion dynamics in the electrolyte can induce spatially asymmetric potential in the semiconductor, an electromotive force for electricity generation.^[^ ^20^ ^]^ However, understanding and controlling the ionovoltaic effect in semiconducting materials remains imperative since multipronged influences of these dipole potential effects from the electrolyte part to the electrode layer were raised as indispensably investigating areas.^[^ ^16^, ^17^, ^22^ ^]^

In this study, the interfacial potential effect of the surficial molecular layer in the ESS structure was investigated through an ionic diffusion‐induced ionovoltaic transducer (IIT). This ionovoltaic measurement in aqueous conditions could be ideal for interrogating the genuine molecular layer effect in the electrolyte–semiconductor interface by excluding subsidiary effects, such as molecular layer degradation under air‐exposed conditions and triboelectric effect upon sequential solid–liquid contact.^[^ ^23^ ^]^ An effect of SAM in IIT was informed with both a change of self‐assembled molecules, which had opposite dipole potentials, and chemical adsorption of transition metal ions in chemically functionalized SAM.^[^ ^16^, ^17^ ^]^ The corresponding intrinsic potential effect of the molecular layer affecting the semiconductor was investigated with various surface analytic techniques. Interfacial interplays across the ESS structure were analyzed with liquid‐interfacing Hall measurement, and therefore, ion‐chelating on molecular layer was revealed to modulate electrical characteristics at the SAM–semiconductor interface by the intrinsic potential modulation of the molecular layer. This was correlated with the output signal behaviors in IIT upon the ion‐chelating process. Ion‐chelating‐dependent output signal variation in IIT shows a strong potential toward interfacial adsorption‐induced probing devices, such as chemical sensing and interfacial analytic tools.

## 2 Results and Discussions

**Figure 1:**
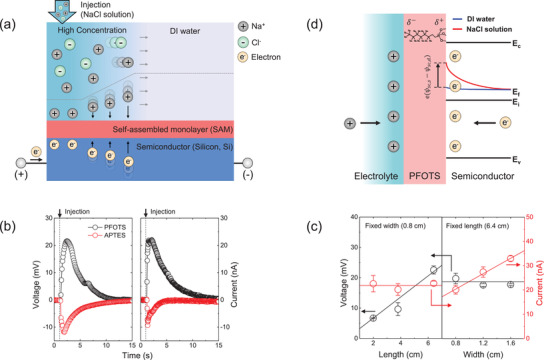
a) A schematic image of ionic diffusion‐induced electricity generation at electrolyte–SAM‐semiconductor (ESS) interface. In the experiments, a highly concentrated solution (NaCl 5 m, 50 µL) was injected into a DI water bath (35 mL). b) Generated voltage and current as a function of time under different SAM conditions (PFOTS and APTES). The NaCl solution, which is a working solution in this experiment, was injected into a water bath at ≈1 s. c) Generated peak voltage and current as a function of device length (*l*, left) and width (*w*, right). In the *l*‐dependent output measurement, *w* was fixed with 0.8 cm. In the *w*‐dependent output measurement, *l* was fixed with 6.4 cm. d) A schematic image of ESS interface (PFOTS) with energy band diagram of n‐Si near a SAM–semiconductor interfacial region. *ψ*_sc,s_ − *ψ*_sc,d_ denotes a difference of potentials under the NaCl solution (*ψ*_sc,s_) and the DI water (*ψ*_sc,d_) near the interfacial region in the semiconducting layer. In Figure 1d, electron energy variation underneath the SAM–semiconductor interface is expressed in the band diagram for intuitive mechanism explanations.

**Figure** **1**a shows a schematic image of electric signal transduction of IIT in ESS structure. The ESS structure is composed of the electrolyte layer, SAM, and silicon (Si) electrode (n‐type, 1000 Ω·cm). The electrode had width (*w*) and length (*l*) of 0.8 and 7 cm, respectively, and SAM, 1H,1H,2H,2H‐perfluorooctyltrichlorosilane (PFOTS), was treated on it for passivation. The PFOTS‐modified device (P‐IIT) was initially soaked in a fixed volume of DI water (a bath with 35 mL), and then a working solution (5 m NaCl 50 µL) was injected to a left end of the electrode, which is connected with a positive pole of a measure unit. As the highly concentrated ions diffuse out, cations adsorb in an electrical double layer (EDL) due to the negative surface potential of PFOTS (Figure S1, Supporting Information), and this adsorption process can be propagated in a lateral direction (Figure 1a).^[^ ^13^, ^24^ ^]^ In this situation, electrons in the semiconducting layer can be attracted toward the SAM–semiconductor interface by an intrinsic dipole potential of the molecular layer, and consistently with the ionic process, this accumulation could be propagated in the lateral direction at the SAM–semiconductor interface.^[^ ^9^, ^20^ ^]^ Hence, electrical outputs could be induced in P‐IIT, as shown in Figure 1b. At first, the generated current and voltage were abruptly evolved and reached an apex, and then they gradually decayed. This could be correlated with the ionic diffusion process at the electrolyte–SAM interface and was interrogated under different cation conditions (1 m of LiCl, NaCl, KCl, and CsCl; Figure S2, Supporting Information). As shown in Figure S2a, Supporting Information, Cs^+^ achieved a highest current, 10.5 nA, among all cations, and these peak values had an order as follows; Cs^+^ > K^+^ > Na^+^ > Li^+^. In addition, Cs^+^ also reached the peak value in the shortest time, 385 ms, and rapidly decayed compared to other ions. This upsurge and attenuation times were also consistent as follows; Cs^+^ > K^+^ > Na^+^ > Li^+^. All these results are matched with an order of ionic diffusivities (Cs^+^ > K^+^ > Na^+^ > Li^+^),^[^ ^25^ ^]^ indicating that the electric signal generation in IIT has a dependence on the ionic diffusion process at the electrolyte–SAM interface. Additionally, this was supported by the dependency of electrical outputs on the concentration gradient of ions in the electrolyte. The electrical measurements of P‐IIT were conducted with varying concentrations of the NaCl working solution (Figure S3, Supporting Information). Similarly with Figure 1b, electrical outputs (both voltage and current) sharply reached an apex and decayed gradually, but their peak values were increased in proportion to the working solution concentration. In this experiment, the highest value was used to explain electric outputs where the effect of the ionovoltaic generation driven by an ionic diffusion is maximized. Since using the electrical signal of peak value is appropriate for a brief explanation, the experimental results will be interpreted based on the maximum value, and 50 µL of 5 m NaCl solution is set as a working solution. To better understand the energy generation in IIT, influences of the molecular layer were further investigated with changing components of the system. (3‐aminopropyl)triethoxysilane (APTES) were treated on IIT instead of PFOTS, and the corresponding device (A‐IIT) outputs were measured under the same condition of P‐IIT. The peak voltage and peak current were 21.6 mV and 21.9 nA in P‐IIT (Figure 1b). As compared to them, similar but reversed voltage and current profiles were measured in A‐IIT (Figure 1b), and those had maximum peaks of −11.84 mV and −9.35 nA, respectively. This could be due to the reversed surface charge and molecular dipole of APTES against PFOTS. According to Figure S1, Supporting Information, the surface potential of APTES is positive, thereby inducing anion adsorption in EDL.^[^ ^26^ ^]^ The dipole potential effect of APTES was also opposite to that of PFOTS, as shown in Figure S4, Supporting Information (UV photoelectron spectroscopy, UPS).^[^ ^27^, ^28^ ^]^ This can indicate that as a similar process with the cation–electron interplays in the PFOTS‐containing ESS, the anion adsorption at APTES could expel electrons (or attract holes) at the SAM–semiconductor interface. This could be the origin of the reversed electrical outputs on A‐IIT. Next, an influence of the Si electrode dimension was also interrogated with P‐IIT. The electrical measurements on the device were conducted with different widths and lengths, as shown in Figure 1c. At the device with fixed width (*w* = 0.8 cm), the peak voltage increased linearly (6.7 to 22.5 mV) with increasing length (*l* = 2 to 6.4 cm), whereas the peak current showed negligible changes (from 20.5 to 22.7 nA) with an average value of 21.79  ±  1.43 nA. On the contrary, the linear increases of the peak current from 20.1 to 32.9 nA were observed with increasing width from 0.8 to 1.6 cm at the device with fixed length (6.4 cm), but peak voltage had little change (from 19.8 to 17.8 mV) with an average value of 18.39  ± 1.2 mV. To elucidate the electrical behaviors of P‐IIT, resistances of each condition were measured (Figure S5, Supporting Information). Under the fixed *w* (=0.8 cm), the device resistances were increased linearly from 0.368 to 0.806 MΩ with elongating *l* from 2 to 6.4 cm. On the other hand, under the fixed *l* (=6.4 cm), the resistance decreased inversely with rising *w* from 0.864 (*w* = 0.8 cm) to 0.326 MΩ (*w* = 1.6 cm). As abovementioned, the ion adsorption in EDL can propagate laterally at the electrolyte–SAM interface by the ionic diffusion, thereby inducing charge carrier movements at the SAM–semiconductor interface (Figure 1a). This ESS interface can be regarded as a multi‐capacitor model,^[^ ^3^, ^5^, ^9^ ^]^ and details of the corresponding interpretation are explained in Note S1 and Figure S6, Supporting Information. Among those processes, the highest peak values of electrical outputs were considered, and corresponding equations of peak current (*I*_p_) and voltage (*V*_p_) were expressed as follows;

(1) $$I_{p}=\frac{D}{\lambda_{D}}\;\varepsilon_{0}\varepsilon_{\mathrm{SAM}}\frac{w}{d}\left(\psi_{\mathrm{sc},s}-\psi_{\mathrm{sc},d}\right)$$

(2) $$V_{p}=R_{\mathrm{sc}}\;I_{p}$$

In this equation, *D* and *λ*_D_ are diffusion coefficient of adsorbed ion and Debye length, respectively, and *D*/*λ*_D_ denotes an adsorption speed at EDL. *ε*_0_, *ε*_SAM_, and *d* are vacuum permittivity, the relative permittivity of SAM, and a thickness of SAM, respectively. *ψ*_sc,s_ and *ψ*_sc,d_ are potentials of semiconductor underneath the SAM interface under the ion‐adsorbed region (denoted with subscript ‘s’) and un‐adsorbed region (denoted with subscript ‘d’), respectively. *R*_sc_ is the resistance of the semiconductor near the SAM interface. As discussed in Figure S2, Supporting Information, alkali ion species‐dependent *I*_p_ was consistent with a direct correlation between *I*_p_ and *D*, indicating that the transient ionic motions at the ESS interface enabled to flow the charge carriers in the semiconducting layer. And then naturally, this concentrated ion propagation near EDL could induce a driving potential, *ψ*_sc,s_ − *ψ*_sc,d_ (a potential difference between highly concentrated and DI water regions), for the charge carrier flows in the semiconducting layer. As shown in Figures S1,S4, Supporting Information, PFOTS had the negative zeta potentials, relying on the electrolyte concentration and the interfacial dipole potential whose negative pole was positioned at the surface.^[^ ^24^ ^]^ Hence, *ψ*_sc,s_ − *ψ*_sc,d_ of PFOTS containing ESS interface was theoretically analyzed as discussed in Note S2 and Figure S7, Supporting Information, and therefore, *ψ*_sc,s_ could be higher than *ψ*_sc,d_. Eventually, the transition from the DI water‐contacted state to the highly concentrated ion condition at EDL can trigger a shift of energy of charge carriers underneath the SAM–semiconductor interface (from red to blue in Figure 1d), thereby inducing an electric current flow in an external circuit. As APTES has opposite surface charges and dipole potentials (Figures S1,S4, Supporting Information), A‐IIT could have a reversed driving potential compared to P‐IIT. Hence the diametrical electric outputs can be generated by the opposite carrier motions in the semiconducting layer (Figure 1b; Figure S8, Supporting Information). The electrical outputs of IIT can also be affected by the device resistance, which can be correlated to the device dimension (Figure 1c). As shown in the Equation (1), *I*_p_ has a direct correlation with *w*, whereas *V*_p_ can have a direct correlation with *l* as shown in the Equation (S9), Supporting Information, a reduced form of the Equation (2). These results are consistent with Figure 1c. Interestingly, those results also indicated that *R*_sc_ could be a determining factor for *V*_p_, and *V*_p_ of IIT could rely on a voltage drop effect in the semiconducting layer adjoining the SAM interface.^[^ ^9^, ^19^ ^]^

**Figure 2:**
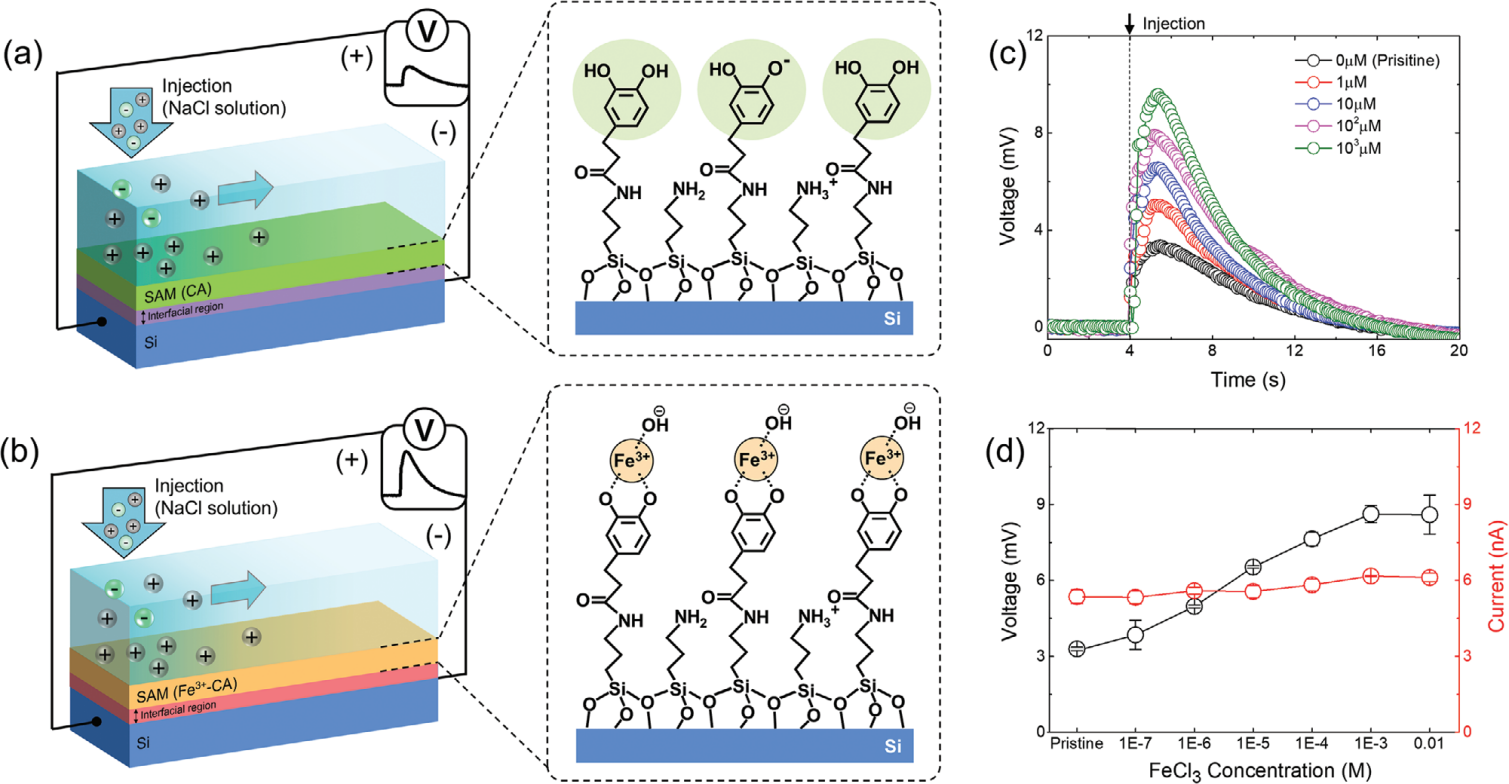
A schematic image of interfacial adsorption‐induced electric output variation in the catechol (CA) group‐modified ionovoltaic device and corresponding interfacial illustrations, which display a) a pristine CA state and b) Fe^3+^‐adsorbed CA (CA‐Fe^3+^) state. Dashed lines in Figure 2b denote coordination bonding between Fe^3+^ and CA ligand atoms. c) Generated voltage as a function of time under different FeCl_3_ concentrations. d) Peak voltage and current as a function of exposed FeCl_3_ concentrations.

To investigate the interfacial molecular effect by ion‐specific adsorption in ESS structure, an ion‐chelating ligand group was introduced at the ESS structure. Catechol group (CA), which can form a coordination complex with ferric ions (Fe^3+^),^[^ ^29^, ^30^ ^]^ was adopted via a chemical modification of APTES as follows; dihydrocaffeic acid (DHCA), which has CA and carboxylic group, was activated by 1‐(3‐dimethylaminopropyl)‐3‐ethylcarbodiimide hydrochloride (EDC·HCl) and N‐hydroxysuccinimide (NHS) to form NHS‐conjugated DHCA (NHS‐DHCA), and then A‐IIT was treated with NHS‐DHCA as shown in Figure S9, Supporting Information. A corresponding schematic image of CA‐modified SAM and its electrical output in CA‐modified device (CA‐IIT) are shown in **Figure** **2**a,c. The prepared CA‐IIT generated *V*_p_ of ≈3.3 mV, and its time‐dependent profile was similar and equivalently polarized with that from P‐IIT. Under the same diffusion process with P‐IIT, this reduced electrical output in CA‐IIT could be originated from a heterogeneous CA‐modified surface, containing both remaining amine groups from APTES and hydroxyl moieties from CA (Figure 2a). After the CA functionalization, the surface zeta‐potential was shifted from highly positive values (Figure S1, Supporting Information; 74.6  ±  0.03 and 57.9  ±  0.1 mV at DI water and 0.1 m conditions, respectively) to slightly negative regions (Figure S1, Supporting Information; −5.12  ±  0.20 and −1.99 ±  0.04 mV at DI water and 0.1 m conditions, respectively) due to an influence of hydroxyl group in CA.^[^ ^31^ ^]^ To investigate the effect of the Fe^3+^‐CA complexation in SAM to IIT, the generated voltage variation upon exposures of FeCl_3_ solution (from 10^−7^ to 10^−2^ m) was monitored as shown in Figure 2b,c. As the exposed solution concentration increased, the magnitude of the generated voltages in the Fe^3+^ adsorbed device (Fe‐IIT) was elevated up to ≈9.6 mV (10^−3^ m) with presenting similar time‐dependent profiles. This indicated that the uniform ion dynamics were applied to operate IITs, and the voltage shifts could be derived from changes of the ion–charge carrier interactions by the Fe^3+^‐CA complexation in SAM. Figure 2d displays both *V*_p_ and *I*_p_ of IITs under different FeCl_3_ concentrations. Consistently with Figure 2c, *V*_p_ was increased in a range of 10^−7^–10^−3^ m and saturated at 10^−2^ m, but *I*_p_ showed a slight increase in that range (from 5.36 nA at pristine state to 6.17 nA at 10^−3^ m; Figure S10, Supporting Information). The saturation of *V*_p_ over 10^−3^ m could be induced from the low pH at this concentration range (Figure S11, Supporting Information), which can interrupt the Fe^3+^‐CA complexation.^[^ ^32^ ^]^ As displayed in the Equation (2), *V*_p_ relies on two factors, *R*_sc_, the resistance of the semiconducting layer near the SAM interface, and *I*_p_. Chemical interactions between CA and Fe^3+^ can rely on surrounding environments, such as pH and their relative quantity. In this experimental condition, Fe^3+^ could favorably form a mono‐complex with surface‐grafted CA due to our pH condition, less than 6 (Figure S11, Supporting Information),^[^ ^32^ ^]^ and a steric effect between them.^[^ ^33^ ^]^ In high FeCl_3_ concentration (over 10^−3^ m), low pHs (Figure S11, Supporting Information) can hamper the Fe^3+^‐CA complexation because the hydroxyl group on CA can preferably exist in a ‐OH form, not in a ‐O^−^ form, which is favorable for the Fe^3+^ complexation. Simultaneously with this, the CA complexed Fe^3+^ could interact with negative hydroxide ions (OH^−^) due to their strong acidity,^[^ ^32^, ^34^, ^35^ ^]^ thereby binding with them in the aqueous interface (Figure 2b). Hence, the Fe^3+^ complexed SAM showed more negative zeta potential than that of pristine CA‐modified surface (Figure S1, Supporting Information). Due to this, more cations can be attracted to the electrolyte–SAM interface. This effect could induce a higher driving potential (*ψ*_sc,s_ − *ψ*_sc,d_) at Fe‐IIT than CA‐IIT, thereby contributing to the slightly increased *I*_p_ depending on the Fe^3+^ complexation (Figure 2d). However, the experimental results in Figure 2d implied that, as compared to *I*_p_, the drastic *V*_p_ shifts could be majorly originated from a change of *R*_sc_ upon the Fe^3+^‐CA complexation in SAM. This could be supported by a gradual elevation of the bulk resistance of the semiconductor by the interfacial Fe^3+^ complexation (Figure S12, Supporting Information; from 1.15 (pristine) to 1.23 (10^−2^ m) MΩ). Hence, this indicated that the Fe^3+^‐CA complexation in SAM also could critically influence the semiconducting layer adjoining the SAM interface. In our previous work, the ionovoltaic generation was confirmed to be regulated by modulations of the interfacial molecular layer (e.g., ion adsorption and molecular interaction).^[^ ^16^, ^17^ ^]^ In these works, physicochemical interactions of the interfacial molecular layer were monitored via an electrolyte–SAM‐metallic electrode interface, and the interfacial molecular dipole effect genuinely determined the electrical behaviors in the device. However, those results indicated that the Fe^3+^‐CA complexation in SAM significantly influenced both the electrolyte and the semiconducting layer, thereby inducing complicated electrical behaviors (increasing *V*_p_ and constant *I*_p_) in the IIT.

**Figure 3:**
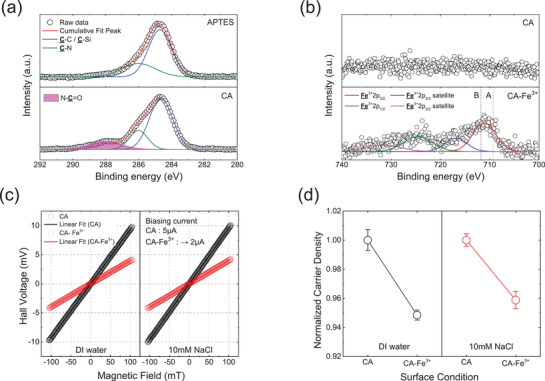
a) C1s XPS spectra of APTES‐modified (top) and Catechol‐modified (bottom) device surface. b) Fe 2p XPS spectra of a pristine catechol‐modified (top) and Fe^3+^‐adsorbed (bottom) surface (exposed to 100 µm FeCl_3_). Dashed lines of A and B denote the positions of Fe 2p_3/2_ peak obtained from Fe^2+^ with O^−^ ligands and Fe^3+^ with Cl^−^ ligands, respectively. c) Hall voltage of the semiconducting layer as a function of a magnetic field at the ESS interface. The Hall measurements were conducted under DI water (left) and 10 mm NaCl (right) conditions. Constant currents of 5 and 2 µA were applied to CA‐modified (black) and CA‐Fe^3+^ (red) devices. The magnetic field was swept from −105 to 105 mT. d) Normalized carrier density as a function of surface states (CA‐modified and CA‐Fe^3+^) under different electrolyte conditions (DI water (left) and 10 mm NaCl (right)).

To identify a dominant factor for the electrical behavior by Fe^3+^‐CA complexation, X‐ray photoemission spectroscopy (XPS) analysis and a liquid‐interfacing Hall measurement are conducted in **Figure** **3**. The chemical composition of each surface was identified through XPS analysis. Figure 3a shows C 1s spectra of APTES‐modified (top) and catechol group‐functionalized (bottom) surfaces. C1s spectrum showed peaks from APTES derivatives, including amine groups (C—N, 286.0 eV) and aliphatic carbon chains (C—C, ≈284 eV).^[^ ^36^ ^]^ However, the peak of amide bond (N—C═O, 288.0 eV), absent in the APTES‐treated surface, was evolved after EDC/NHS reaction as presented in the CA‐modified surface (bottom of Figure 3a), indicating that catechol groups had successfully grafted to the surface. To investigate the chemical states of coordination complex, XP spectra of Fe 2p at the CA‐modified and Fe^3+^ adsorbed surfaces (CA‐Fe^3+^) are shown in Figure 3b. The characteristic doublet peaks of Fe2p_3/2_ and Fe2p_1/2_, which were not found at the CA, exhibited at the CA‐Fe^3+^ showing the presence of iron elements on the surface. As shown in Figure 3b, the binding energy of Fe2p_3/2_ peak (711.1 eV) at the CA‐Fe^3+^ was positioned between A, ferrous ionic state (Fe^2+^) (709.0 eV, Fe_2_SiO_4_),^[^ ^37^ ^]^ and B, ferric ionic state (Fe^3+^) (711.5eV, FeCl_3_).^[^ ^38^ ^]^ This indicated that the chemical state of CA‐Fe^3+^ could be an oxygen group coordinating state, offered by CA, instead of counter‐anion (Cl^−^). Iron ion in the solution of FeCl_3_∙6H_2_O mainly exists in the form of [Fe(H_2_O)_4_Cl_2_]^+^, which has two Cl^−^ ligands.^[^ ^39^, ^40^ ^]^ During the Fe^3+^‐CA complexation, Cl^−^ ligands of iron ion are exchanged to O^−^ ligands from CA. This was supported by the XPS spectrum of Cl 2p at the CA‐Fe^3+^ where no peak was evolved (Figure S13, Supporting Information). According to the crystal field theory, ‐O^−^ group is a stronger field ligand than Cl^−^ and then forms a large splitting of d‐orbitals in a coordination complex in which the outermost electrons of Fe^3+^ could be mostly in t_2g_ orbitals.^[^ ^41^ ^]^ Therefore, the binding energy of Fe^3+^ with ‐O^−^ ligands can be positioned lower than that with Cl^−^ because electrons might be easily eliminated in a lower spin complex with ‐O^−^ ligands. To investigate the influence of the Fe^3+^‐CA complexation to the SAM–semiconductor interface, a liquid‐interfacing Hall measurement was conducted (Figure 3c,d). This measurement is a novel analysis to interrogate the ion‐semiconductor interaction at the solid–liquid interface.^[^ ^20^ ^]^ The measurements were conducted on the CA‐IIT and Fe‐IIT submerged in DI water and 10 mm NaCl solution, and optimal biasing currents were applied for each device due to their different resistances (CA: 5 µA, CA‐Fe^3+^: 2 µA, Experimental Section). Hall voltage (*V*_H_) was measured by applying an external magnetic field in a normal direction to the sample surface (−105 to 105 mT), and slopes are calculated by linear regression in Figure 3c. From this, the number density of charge carriers (*n*) in each condition was calculated based on Hall effect (Figure S14, Supporting Information),^[^ ^42^ ^]^ and to analyze the influence of the Fe^3+^‐CA complexation to the SAM–semiconductor interface, they were normalized with respect to that in the CA‐IIT. In both highly concentrated (10 mm NaCl) and DI water conditions (Figure 3d), the normalized carrier density of the semiconductor was decreased from 1 to 0.96 after the Fe^3+^‐CA complexation (details are denoted in the Experimental Section). Although *n* from the Hall effect presents a bulk characteristic of the semiconductor, this *n* reduction after the Fe^3+^ chemisorption at SAM suggested that the interfacial molecular effect at the ESS structure can be critically influential to the adjoining semiconducting region.^[^ ^20^ ^]^ These results could provide significant evidence for the electrical behaviors of the CA‐IIT and Fe‐IIT (Figure 2d). Consistently with Figure S12, Supporting Information, the Fe^3+^‐CA complexation at SAM could deplete charge carriers at the SAM–semiconductor interface by its dipole potential shift. This is in accordance with the positive work function shift of the CA‐IIT after binding Fe^3+^ on them (Figure S4, Supporting Information). Hence, *R*_sc_ can be increased, thereby escalating the voltage drop effect (the increase of *V*_p_ in Figure 2d) at the SAM–semiconductor interface. Although there should be detailed understandings for surficial molecular effect, the Fe^3+^ complexation‐induced molecular dipole shift (Figure 3d; Figure S4, Supporting Information) might be resulted from extra‐coordinations of negative hydroxide ions in the Fe^3+^‐CA complexes (Figure 2b) at the electrolyte‐contacted state. In addition, the weak variation of *I*_p_ upon the Fe^3+^ complexation could be induced from this charge carrier depletion effect, which can compete with the increase of surface charges due to the bound hydroxide ions in the Fe^3+^‐CA complexes (Figure 2b).

**Figure 4:**
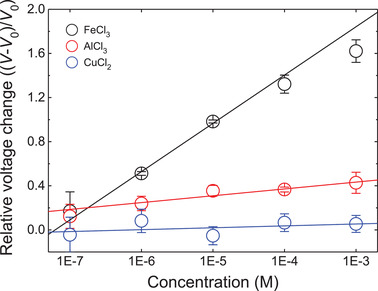
Relative peak voltage changes as a function of metal ion solution concentrations. Here, *V*_0_ denotes a peak voltage at the pristine (CA‐modified) state. Lines in Figure 4 denote a semi‐log plot obtained by a linear regression of each condition as follows; (*V* − *V*_0_)/*V*_0_ =  *a*(log *c*) + *b*, where *a* and *b* are a slope and an intercept of the plot, respectively. Detailed information is described in the Experimental Section. According to the fitting, each slope of FeCl_3_, AlCl_3_, and CuCl_2_ are 2.754 (*R* ^2^ = 0.997), 0.670 (*R* ^2^ = 0.929), and 0.021 (*R* ^2^ = 0.009), respectively.

To further investigate the electrical outputs of IIT depending on the ion‐ligand complexation, different metal ions, Fe^3+^, Al^3+^, and Cu^2+^ (Cl^−^ as a same counterion), were exposed to IIT surface (**Figure** **4**). The experiments were conducted in the same method with the FeCl_3_ test shown in Figure 2. Output voltages depending on each ion concentration were measured, as shown in Figure S15, Supporting Information, and presented as relative peak voltage changes ((*V* − *V*_0_)/*V*_0_, where *V*_0_ is a peak voltage at the pristine CA‐IIT). Interestingly, upon the metal ion exposures, the output voltages from IIT were increased in all cases, but their magnitudes were varied depending on each ion species. From linear regressions of these output voltage changes, slopes in each plot were obtained as the following order; Fe^3+^: 2.754 (*R* ^2^ = 0.997) > Al^3+^: 0.670 (*R* ^2^ = 0.929) > Cu^2+^: 0.021 (*R* ^2^ = 0.009). As mentioned in a previous section, Fe^3+^ can form the most stable complex with the CA. Hence, this could be consistent with a radical voltage variation of Fe^3+^ shown in Figure 4, even though CA was anchored in the solid surface. In the Al^3+^ case, the gradual voltage increase with the high correlation (*R* ^2^ = 0.929) was observed, but Cu^2+^ showed a negligible change with a faint correlation (*R* ^2^ = 0.009). Al^3+^ and Cu^2+^ also could form a mono‐complex with the catechol group, but its stability constants were much lower than the Fe^3+^‐CA mono‐complex (a stability constant order of metal ion‐catechol mono‐complex; Fe^3+^ > Al^3+^ > Cu^2+^; Table S1, Supporting Information).^[^ ^29^ ^]^ In addition to this, an acidity difference between these ions also could contribute to this voltage variation (Table S1, Supporting Information). Especially, the drastic voltage behavior in Fe^3+^ could be attributed to a higher acidity of Fe^3+^, forming more OH^−^ bound state than other ions in an aqueous state. As discussed in the previous section, the more OH^−^ binding to the transition metal ion‐CA complex could induce a much larger interfacial molecular dipole shift, thereby triggering a higher depleting effect at the SAM–semiconductor interface. Although in‐depth understandings are required to the interfacial state of the coordination complex and their interactions within the ESS interface, the voltage behaviors shown in Figure 4 can represent an interesting mutual interaction between the ESS interfaces depending on influences of transition metal–ion‐interfacial molecular complexes. Therefore, this result shows that CA have an applicability as novel ion sensor for specific ferric ion detection by observing significant signal change by adsorption compared to other metal ions.

## 3 Conclusion

In summary, the ionovoltaic mechanism at the ESS interface and the corresponding influences of the interfacial molecular layer were interrogated via the chemical controls of the molecular layer. The ion–charge carrier interplays originated from the interfacial dipole potential were verified in IIT. In addition, influences of the interfacial molecular layer to this system were demonstrated via the amine group chemistry‐based CA modification and the ion‐ligand complexation therein. Both IIT and the liquid‐interfacing Hall measurement consistently proved that the Fe^3+^‐CA complexation at the electrolyte–semiconductor interface could deplete the charge carrier density at the semiconducting layer adjacent to the interface, thereby suggesting an impact of the surficial molecular layer to an energy level of charge carriers under the electrolyte‐interfacing condition. Unlike conventional surface analytic techniques, which require high vacuum conditions or can be disturbed by high noise signals (originated from the energetics of water molecules), these results indicated that the concept of this IIT using the ionovoltaic mechanism could be an interface‐sensitive measurement and applicable in analyzing the various electrolyte–semiconductor interface.^[^ ^43^, ^44^ ^]^ In addition, with diverse surface functionalization chemistry, this ion dynamics‐driven transducing system could be utilized as effective interfacial or molecular probing device.

## 4 Experimental Section

### Fabrications of PFOTS‐Modified Device (P‐IIT) and APTES‐Modified Device (A‐IIT)

Silicon (Si) wafer (n‐type, 1000 Ω·cm, MTI Corporation) was cut into 0.8 × 7 cm^2^ and ultrasonicated sequentially with detergent, DI water, acetone, and isopropyl alcohol (IPA) for 15 min each. O_2_ plasma (0.6 torr, 100 W) was treated on the Si substrate for 30 s to form silanol groups (Si‐OH) for the deposition of a SAM. a) P‐IIT: PFOTS was deposited by chemical vapor deposition. The O_2_ plasma‐treated Si was placed in a desiccator with a slide glass containing 30 µL PFOTS under vacuum for 10 min at room temperature. The PFOTS‐treated Si was rinsed with IPA and annealed at 120 °C for 20 min to remove untreated PFOTS. b) A‐IIT: APTES was deposited by liquid phase dipping. The O_2_ plasma‐treated Si was dipped in 2% v/v APTES in toluene at 90 °C for 1 h. The APTES‐treated Si was sonicated with toluene for 1 min, rinsed with toluene and ethanol sequentially, and dried under N_2_ flow.

### Fabrication of Catechol Groups Modified IIT (CA‐IIT) and Fe^3+^ Adsorbed CA‐IIT (Fe‐IIT)

DHCA was chemically reacted with amine groups of A‐IIT by EDC/NHS reaction as shown in Figure S9, Supporting Information. DHCA was activated to NHS‐DHCA by mixing 80 mm EDC∙HCl, 40 mm NHS, and 20 mm DHCA in 0.1 m 2‐(N‐Morpholino)‐ethanesulfonic acid hydrate aqueous solution for 15 min. The A‐IIT was dipped in the NHS‐DHCA solution for 1 h and rinsed with DI water, and then CA‐IIT was prepared. The Fe^3+^ solutions of each concentration were prepared by dissolving FeCl_3_·6H_2_O salts in DI‐water. Then, CA‐IIT was exposed to the solution for 10 min and rinsed with DI water several times. Then, Fe^3+^ adsorbed device (Fe‐IIT) was prepared.

### Surface Characterizations

The surface chemical states of the devices were characterized by XPS (NEXSA, Thermofisher). To characterize a potential above the surfaces, zeta potential measurement (SurPASS 3, Anton Paar) was performed under DI water and a 10 mm NaCl solution atmosphere. To characterize potential difference at the SAM, UPS (NEXSA, Thermofisher) was conducted. The Work function of each device was obtained, and its shift referred to bare‐Si was calculated. The samples measured in XPS, UPS, and zeta potential were prepared in Si substrates with the same method of preparing the devices.

### Electrical Characterization of the Devices

A Keithley 2182A nanovoltmeter and 6485 picoammeter were used to measure the output voltage and current of the device, respectively. Both lateral ends of the exposed Si electrode at the prepared device were connected to the wire and sealed with an epoxy resin to prevent leak current by contacting with water. Then, the device was placed on the center of a round Petri dish bath, and 35 mL of DI water was filled into the bath to soak the device. The probe of the electrical measurement unit was connected to one side where an ionic solution was injected, and the ground electrode was connected to the other side. A micropipette tip filled with 5 m NaCl 50 µL was vertically brought to the aqueous bath surface where the device was connected with the probe using a custom‐built motion controller. During the ionic diffusion process, an early stage peak, which was induced by initial contact of the ionic solution with the DI water‐SAM interface, could be observed. Sequentially, the attenuating signal originated from the lateral ionic diffusion could be monitored. During the lateral diffusion, there was a possibility of generating irregular signals in the attenuating profile, which might be derived from the random ionic motion in the water bath. Hence, to reduce this effect, strict control of the working solution injection (e.g., automatic motion controller) was necessary for the experiment.

### Characterization of Semiconductor Property Using a Liquid‐Interfacing Hall Measurement

The edges of the electrode of each fabricated device were sealed by the epoxy resin, including four terminals which were connected through wires on a sample (width × length = 10 × 10 mm^2^), the lines of current and voltage were cross‐connected to each other. Each sample was placed in a 15 mL conical tube containing 4 mL DI water and ionic solution (10 mm NaCl) to completely submerge it, respectively. The direct current constantly biased to the current terminal while applying an external magnetic field in the direction on the vertical plane of the sample was 5 µA for CA‐IIT and 2 µA for Fe‐IIT, and then the Hall voltage with the sweeping magnetic field (−105 to 105 mT) was measured with Keithley 197 Auto‐ranging microvolt DMM. The frequency of the function generator to measure the number of Hall voltage data and the resolution was set to 1.7 mHz, and the voltage values were obtained at 0.45 mT intervals.

### Statistical Analysis

In Figures 1c,2d, each data point is obtained from an average of three independent measurements. In Figures 2,3d, different samples are measured, and an average value of them was denoted in the graph. In Figure 4, each data point is obtained from an average of three independent measurements, and linear regression was conducted to each ion condition with a semi‐log plotting form ((*V* − *V*_0_)/*V*_0_ =  *a*(log *c*) + *b*). For Fe^3+^ and Al^3+^, high linearity was obtained (*R* ^2^ = 0.997 (Fe^3+^) and 0.929 (Al^3+^)), whereas Cu^2+^ showed a negligible correlation (*R* ^2^ = 0.009). For slope values (*a* in the fitting equation), *p*‐values of Fe^3+^, Al^3+^, and Cu^2+^ were 3.52 × 10^−5^, 5.25 × 10^−3^, and 3.83 × 10^−1^ (for alpha value of 0.05), respectively, indicating high reliabilities of the Fe^3+^ and Al^3+^ results and a low correlation of the Cu^2+^ result. This was consistent with the linearity results. All plotting and analysis were conducted with Origin 9.0.

## Conflict of Interest

The authors declare no conflict of interest.

## Supporting information

Supporting Information

## Data Availability

The data that supports the findings of this study are available in the Supporting Information of this article.
