# Supplementary material for: Ionic Diffusion‐Driven Ionovoltaic Transducer for Probing Ion‐Molecular Interactions at Solid–Liquid Interface
Source: Adv Sci (Weinh). 2021 Oct 31;9(1):2103038. doi: 10.1002/advs.202103038 (PMC8728816; doi:10.1002/advs.202103038)
Supplement: Supplementary file 1 — Supporting Information [file ADVS-9-2103038-s001.pdf]

WILEY-VCH

© 2021 Wiley-VCH GmbH

ADVANCED SCIENCE
Open Access

Supporting Information

for *Adv. Sci.*, DOI: 10.1002/advs.202103038

Ionic diffusion-driven ionovoltaic transducer for probing ion-molecular interactions at solid-liquid interface

*Junghyup Han, Sun Geun Yoon, Won Hyung Lee, Huding Jin, Yong Hyun Cho, and Youn Sang Kim**

Supporting Information

Ionic diffusion-driven ionovoltaic transducer for probing ion-molecular interactions at solid-liquid interface

*Junghyup Han, Sun Geun Yoon, Won Hyung Lee, Huding Jin, Yong Hyun Cho, and Youn Sang Kim**

Supporting Note 1. Derivation of device output equation

When the PFOTS-treated Si device (P-IIT), which has certain width (w) and length (l), was immersed in DI water, charging of cations at the PFOTS-electrolyte interface can be induced by the diffusion process (Aqueous phase in Figure S6a). This interfacial charging is driven by an intrinsic potential difference emanating from PFOTS, and therefore, it is reasonable to regard the electrolyte-SAM-semiconductor interface as capacitors (from C_1 to C_n) at unit areas.[1–3] Hence, the same number of electrons, equivalent to an amount of the adsorbed ions, can be accumulated at the PFOTS-semiconductor interface. In the circuit of Figure S6a, R'_1~R'_n and R_1~R_{n-1} represents resistance of a unit resistor in aqueous phase and in semiconductor near the interface, respectively, between each capacitor, and $R_1 + R_2 + \cdots + R_{n-1} = R_{sc}$, which is a resistance of the semiconductor near the SAM interface.

After an injection of highly concentrated solution, an ionic current (I_i) by the interfacial ion charging (at C_1) can drive an equal amount of electrical current in the semiconductor ($I + I_s$, where I and I_s are electrical currents flowing through an external load and the semiconductor, respectively). Sequentially, lateral diffusion process (serial switch on in Figure S6a) enables the concentrated ions to be charged at C_n or dissipated toward bulk part, thereby inducing both equivalent charging of electrons at C_n and corresponding electrical current (I and I_s) at the electronic circuit. However, as compared to the first interfacial charging (at C_1), which is driven by the diffusion under an initial concentration of the electrolyte, the amount of adsorbed ions at the posterior capacitors (from C_2 to C_n) could become reduced due to an ion dissipation toward a bulk part. In addition, during the ion dissipation toward the low concentration region (DI water), there is a possibility of occuring irregular ionic fluxes in the

water bath. Hence, as shown in Figure 1b, the electric output could have an apex at the first charging (C_1) and then be irregularly attenuated as time goes by. Hence, as shown in Figure 1b, the electric output could have an apex at the first charging (C_1) and then be decayed as time goes by. Herein, the apparent peak of electric outputs is considered as a major signal, and a corresponding simplified equivalent circuit is displayed at Figure S6b. In the circuit of Figure S6b, a relation between each current flow can be expressed by

$$I = I_i - I_s \tag{S1}$$

Voltage measured in external voltammeter (V) can be presented as follows;

$$\mathrm{V} = I_s R_{sc} - I R_{ext}$$

(S2)

, where R_{ext} is a resistance in external circuit including contact resistances and assumed to be much smaller than R_s. I_i by the solution injection can be expressed with dQ/dt, where Q is an amount of ions (electrons) adsorbed (accumulated) at the electrolyte-SAM-semiconductor interface. Hence,

$$I_i = \frac{dC_1}{dt} \Delta\psi \tag{S3}$$

, and $\Delta\psi$ is a potential difference between electrolyte and semiconductor, which are in the vicinity of the SAM interface. This is equivalent to a potential difference between underneath the ion-adsorbed interface (denoted with subscript‘s’) and un-adsorbed (DI water) interface (denoted with subscript‘d’) in the semiconducting layer. Hence, the equation (S1) can be denoted with both the equation (S2) and the equation (S3) as follows;

$$I = -\frac{1}{R_{sc}} \left(V - R_{sc} \frac{dC_1}{dt} \left(\psi_{sc,s} - \psi_{sc,d} \right) \right)$$

(S4)

$\psi_{sc,s}$ and $\psi_{sc,d}$ denote a potential of semiconductor near the SAM interface under the ion-adsorbed region and un-adsorbed region, respectively. Due to the diffusion-induced capacitive charging (Figure 1a), dC_1/dt can be expressed with an adsorption speed in electric double layer (D/λ_D, where D and λ_D are a diffusion coefficient of adsorbed ion and Debye length, respectively) as follows[4];

$$\frac{dC_1}{dt} = \frac{D}{\lambda_D} \varepsilon_0 \varepsilon_{SAM} \frac{w}{d} \tag{S5}$$

Herein, ε_0, ε_{SAM}, and d are vacuum permittivity, the relative permittivity of SAM, and a thickness of SAM, respectively. λ_D is assumed as DI water condition (equivalent to an initial state in the experiment). Thus, the equation (S4) can be expressed as follows;

$$I = -\frac{1}{R_{sc}}\left(V - R_{sc}\frac{D}{\lambda_D}\varepsilon_0\varepsilon_{SAM}\frac{w}{d}\left(\psi_{sc,s} - \psi_{sc,d}\right)\right)$$

(S6)

In a short-circuited condition, *V*=0, and then

$$I_p = \frac{D}{\lambda_D}\varepsilon_0\varepsilon_{SAM}\frac{w}{d}\left(\psi_{sc,s} - \psi_{sc,d}\right) \quad \text{(S7)}$$

In an open-circuited condition, *I*=0, and then

$$V_p = R_{sc}\frac{D}{\lambda_D}\varepsilon_0\varepsilon_{SAM}\frac{w}{d}\left(\psi_{sc,s} - \psi_{sc,d}\right) \quad \text{(S8)}$$

, which is equivalent to $V_p = I_p \times R_{sc}$. The equation (S8) can be expressed with a sheet resistance of semiconductor (R_{sq}) by considering the extrinsic factors as follows.

$$V_p = R_{sq}l\frac{D}{\lambda_D}\frac{\varepsilon_0\varepsilon_{SAM}}{d}\left(\psi_{sc,s} - \psi_{sc,d}\right) \quad \text{(S9)}$$

Supporting Note 2. Interpretation of the ESS interface

In the equation (S7), $\psi_{sc,s} - \psi_{sc,d}$ is a driving force of charge carrier flows near the SAM-semiconductor interface. As assumed in Supporting Note 1, the electrolyte-SAM-semiconductor interface can be regarded as the capacitor, of which electrodes have different potential screening capabilities. As shown in Figure S7a,b, an equal quantity of charges (ions and electrons) can be accumulated at the SAM (PFOTS) interface, which can have a interfacial charge density of σ_{SAM}. Hence, in both high (subscript 's') and low (subscript 'd') concentration regions, an adsorbed ion density at Stern layer ($\sigma_{st,s}$ and $\sigma_{st,d}$, respectively) can be equivalent to the charge density (per unit area) at the SAM-semiconductor interface ($\sigma_{sc,s}$ and $\sigma_{sc,d}$, respectively) ($\sigma_{st} = \sigma_{sc}$). As σ_{sc} can be expressed as

$$\sigma_{sc} = qNW \quad \text{(S10)}$$

, where q, N, and W are an electron charge, a dopant density, and a width of space charge region,

respectively.[5] qN is constant throughout the semiconducting layer. From the zeta potential (ζ) measurement, $\zeta_s < \zeta_d$ indicated that $\sigma_{st,s} > \sigma_{st,d}$ (Figure S1a). Hence, in the high concentration region, larger $\sigma_{sc,s}$ than $\sigma_{sc,d}$ can induce a relatively wider W_s than W_d (Figure S7a,b). ψ_{sc} can be shown with σ_{sc} and W as follows;[5]

$$\psi_{sc} = \frac{qNW^2}{2\varepsilon_0\varepsilon_{sc}} = -\frac{\sigma_{sc}W}{2\varepsilon_0\varepsilon_{sc}}$$

(S11)

ε_{sc} is a dielectric constant of the semiconductor. Hence, as shown in Figure S7c,d, $\psi_{sc,s}$ can be much larger than $\psi_{sc,d}$ and spanned to deeper region in the semiconductor. Therefore, in the PFOTS case, $\psi_{sc,s} > \psi_{sc,d}$ and this was consistent with the current direction in Figure 1b. On the other hand, in the case of APTES, the anion adsorption at the electrolyte-SAM interface could induce repulsion of electrons (or accumulation of holes) at the highly concentrated region due to its opposite dipole from PFOTS (Figure S8), thereby driving reversed potential direction in semiconducting layer underneath the SAM interface. Hence, A-IIT can have a reversed pole in its output signals as shown in Figure 1b.

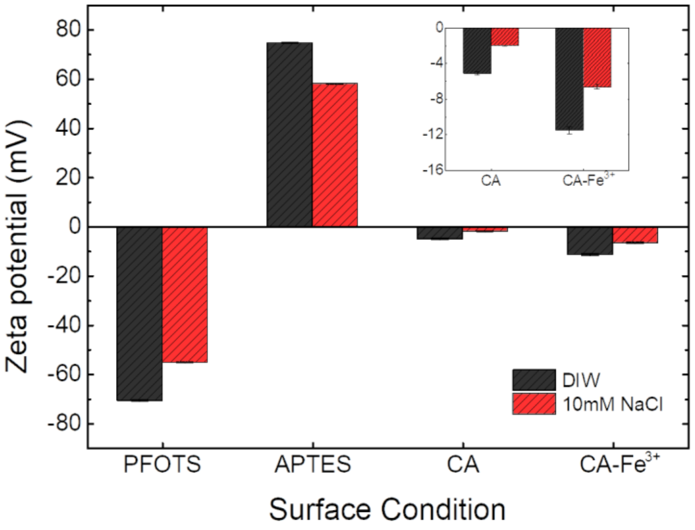

Figure S1. Zeta potential (ζ) under different surface conditions.

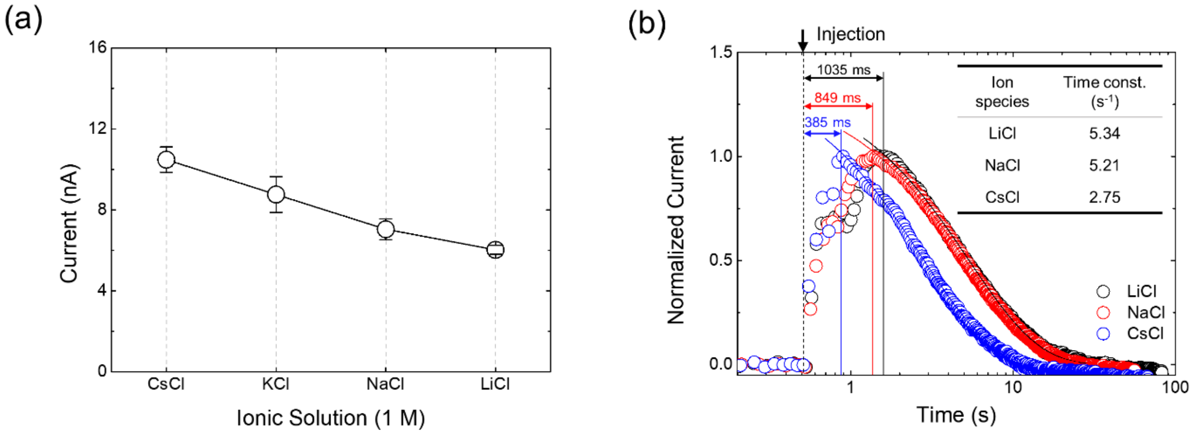

Figure S2. (a) Generated peak current under different alkali ion solutions (working solution). 1 M of each ionic solution was tested in the experiment. (b) Normalized current curves as a function of time under 1 M of LiCl, NaCl, and CsCl. A table in Figure S2b denotes a time constant obtained from fitting of each decaying profile.

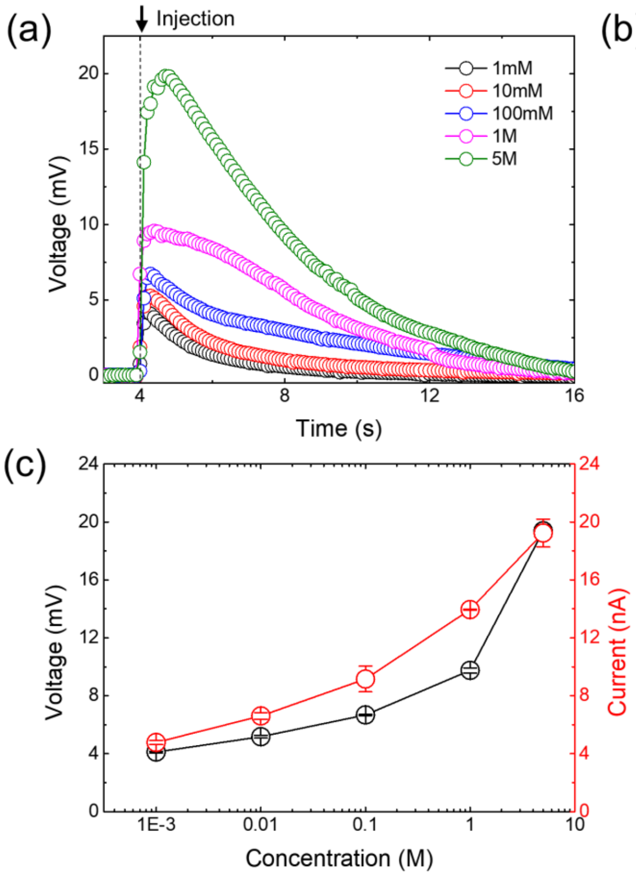

Figure S3. Generated (a) voltage and (b) current as a function of time under different working solution concentrations (NaCl). (c) Peak voltage and current as a function of the working solution concentration.

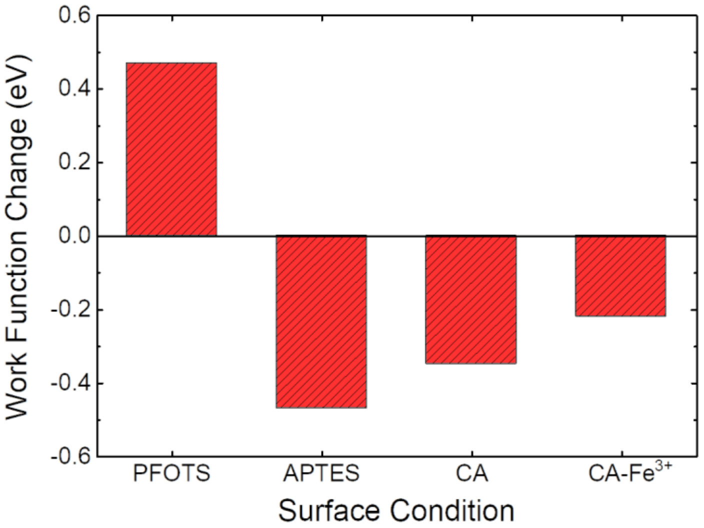

Figure S4. Electron energy changes of Si under different surface conditions. Each measured value was subtracted with a work function of bare Si as a reference (3.23 eV).

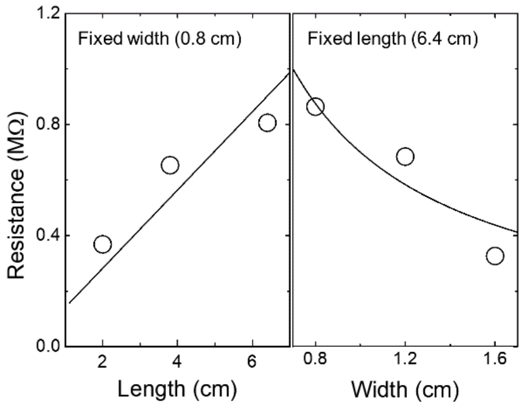

Figure S5. Measured resistance as a function of device dimensions ((left) length and (right) width)

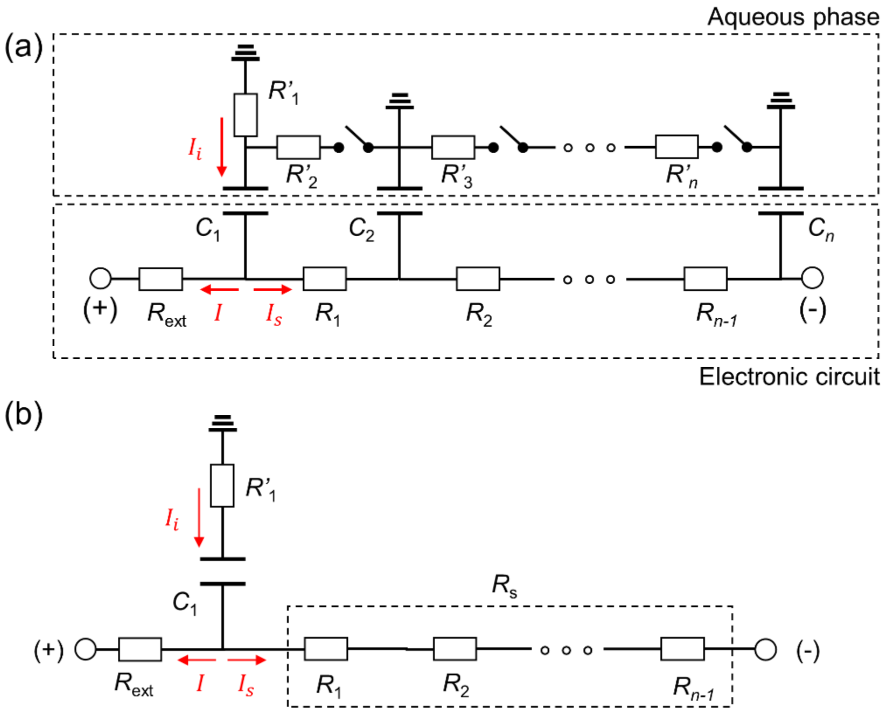

Figure S6. (a) An equivalent circuit of the transducer. When I_i, an ionic current, is induced by the solution injection, an interfacial charging of ions can attract electrons at a capacitor (C_1), thereby generating electrical currents, I and I_s (electrical currents flow through an external load (R_{ext}) and an internal resistor (R_{sc}) near the SAM interface, respectively); $R_{sc} = R_1 + R_2 + \cdots + R_n$. After the first interfacial charging at C_1, electrolyte can be diffused in aqueous phase (switch on), and therefore, drive both sequential interfacial charging (C_{n+1}) and

dissipation towards bulk phase, simultaneously. R'_{n+1} is an resistance in each ionic diffusion. (b) A simplified equivalent circuit that is considering the first interfacial charging event.

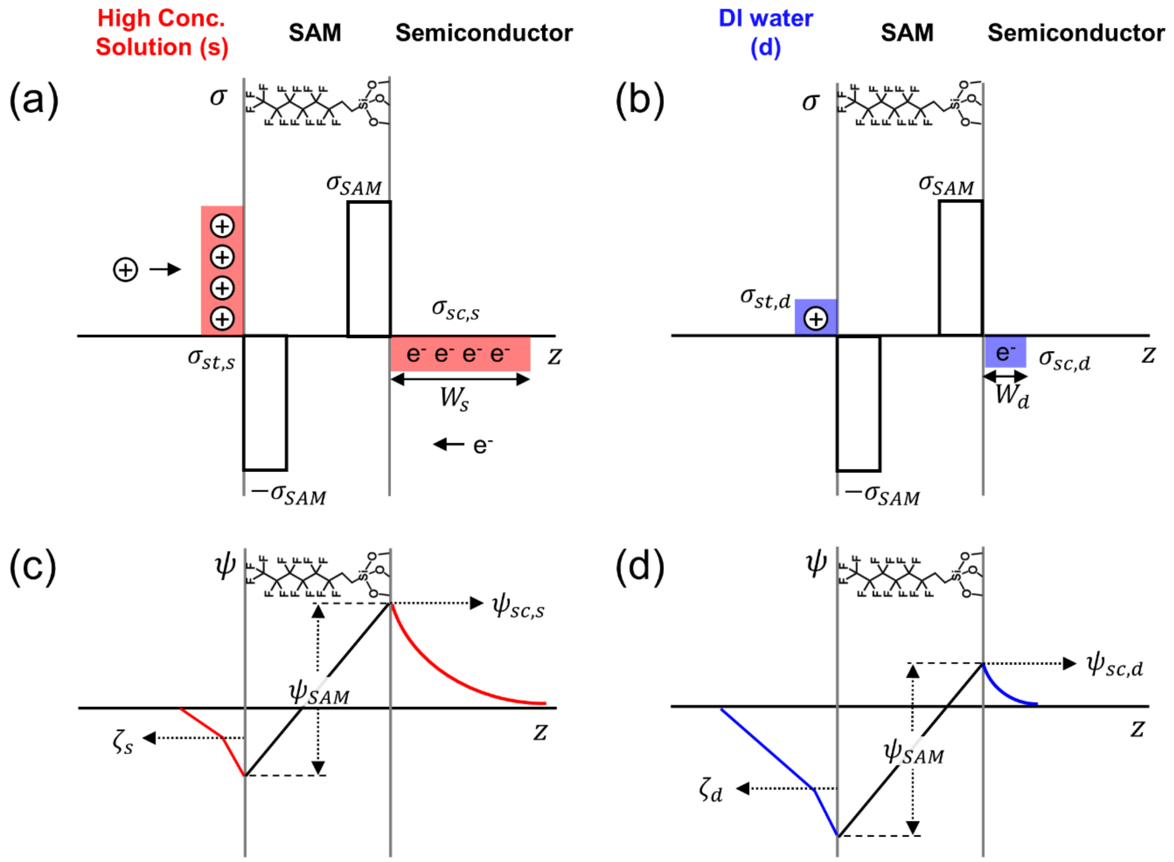

Figure S7. (a, b) Space charge (σ) models of electrolyte-SAM-semiconductor (ESS) interface under (a) high concentration solution and (b) DI water conditions. (c,d) Corresponding vertical potential profiles across the ESS interface under (c) high concentration solution and (d) DI water conditions. Here, the ESS interface can be regarded as a capacitor containing an intrinsic potential difference (ψ_{SAM}), which is derived from the dielectric layer (SAM). σ_{st}, σ_{sc}, and σ_{SAM} are charge density at Stern plane, near the SAM-semiconductor interface, and at SAM interface, respectively. ψ_{sc}, w, and ζ are a potential near the SAM-semiconductor interface, a width of space charge region, and zeta potential, respectively. The subscript 's' and 'd' denote an electrolyte condition of each variable.

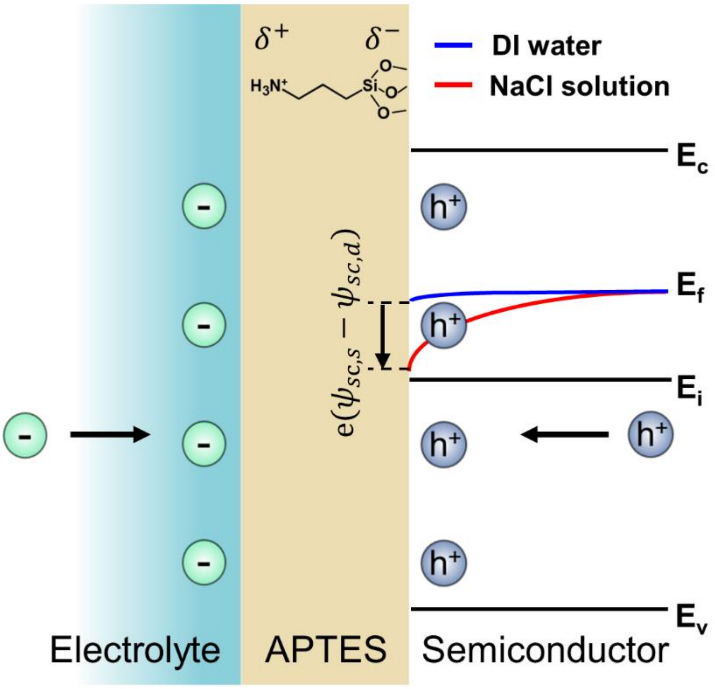

Figure S8. A schematic image of ESS interface (APTES) with energy band diagram of n-Si near a SAM-semiconductor interfacial region. Herein, only electron energy variation underneath the SAM-semiconductor interface was expressed in the band diagram for intuitive mechanism explanations. In the figure, an charge carrier response, which can be induced by anion adsorption at the electrolyte-SAM interface, was described as an accumulation of holes, equivalent to electron repulsion, at the SAM-semiconductor interface.

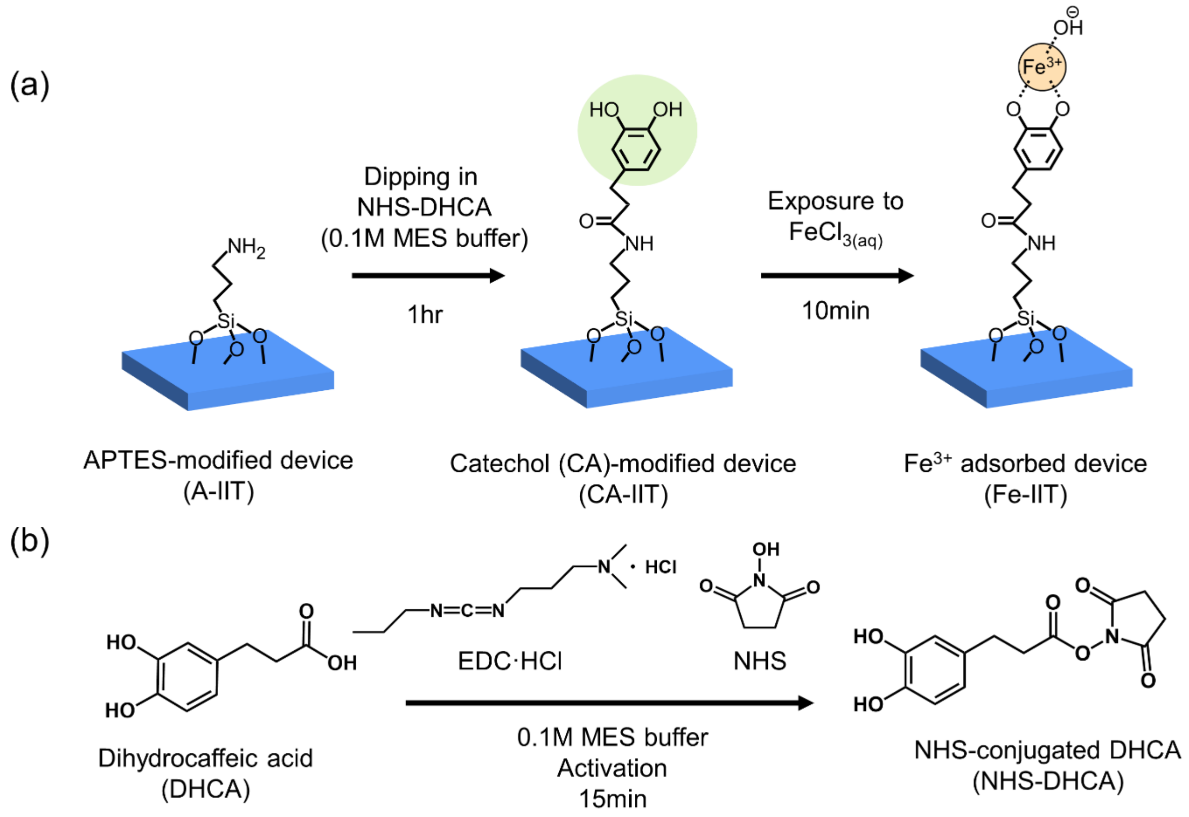

Figure S9. Experimental process to prepare catechol-modified surface and Fe^{3+} adsorbed surface. (a) Surface functionalization of A-IIT by NHS ester reaction and the exposure of CA-IIT to $FeCl_3$ solution. (b) Activation of DHCA to NHS-DHCA

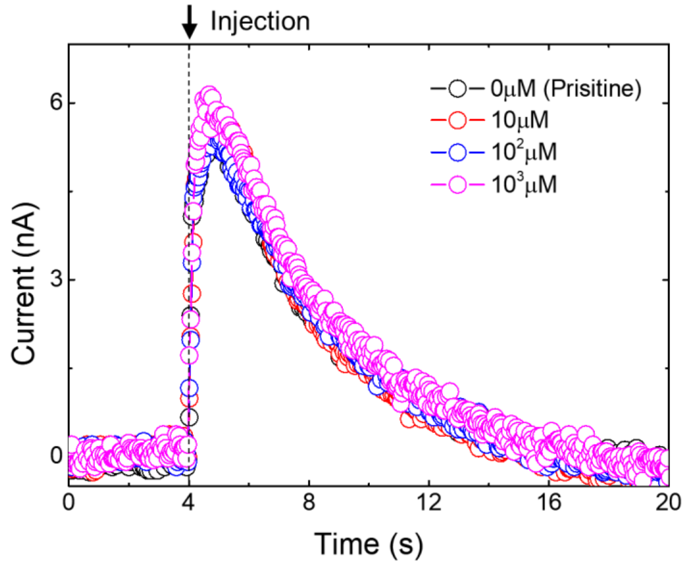

Figure S10. Generated current as a function of time under different $FeCl_3$ concentrations.

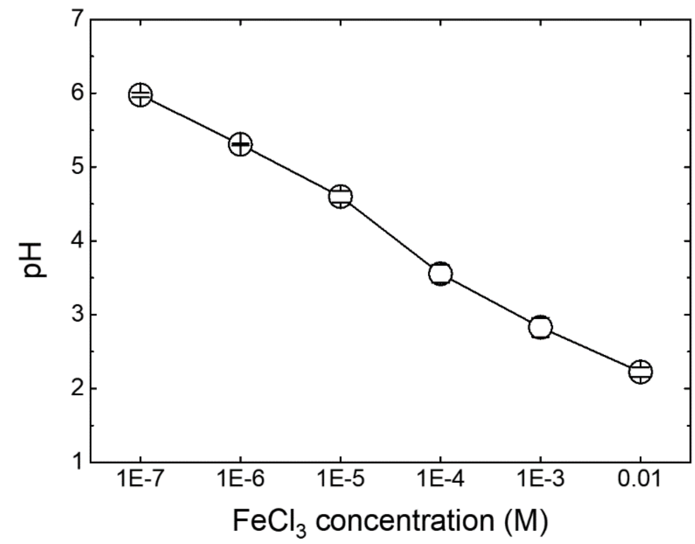

Figure S11. The pH of $FeCl_3$ solution as a function of concentration.

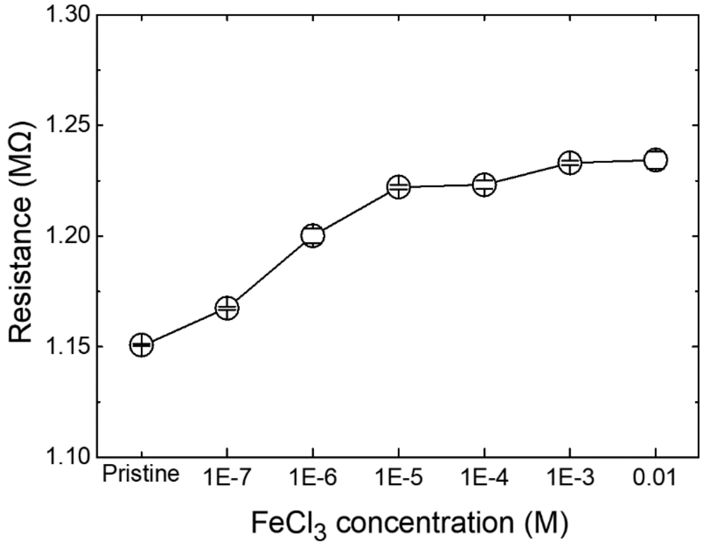

Figure S12. Device resistance as a function of exposed $FeCl_3$ concentrations. Resistances in each condition was obtained from a slope of current-voltage curves, which were measured at both terminals.

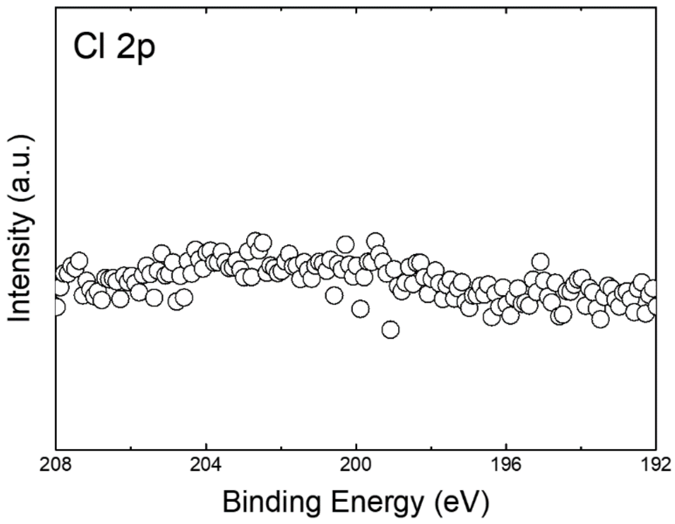

Figure S13. Cl 2p XPS spectrum of Fe^{3+}-adsorbed CA surface when exposed to 100 μM $FeCl_3$

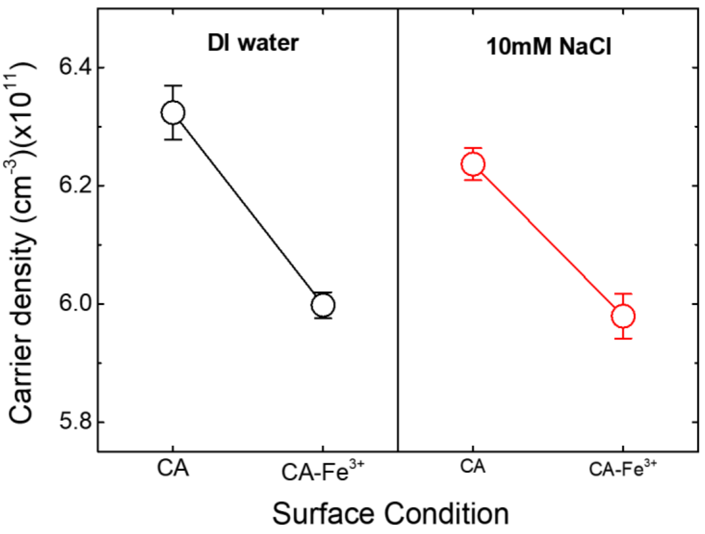

Figure S14. Carrier density as a function of surface states (CA-modified and CA-Fe^{3+} states) under different electrolyte conditions ((left) DI water and (right) 10 mm NaCl).

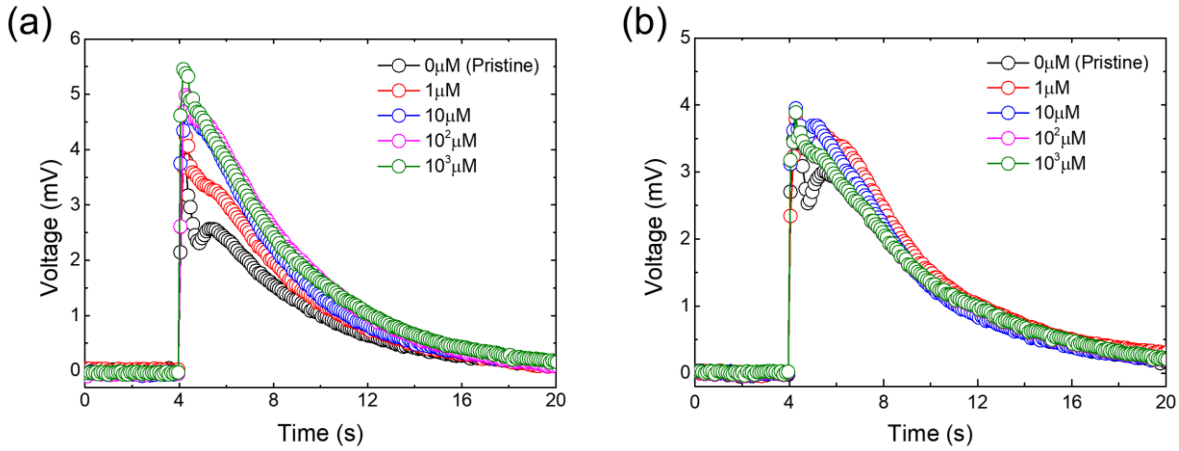

Figure S15. Generated voltage as a function of time under different (a) $AlCl_3$, (b) $CuCl_2$ concentrations.

Table S1. Stability constant (log K) of mono-complex formation of metal ion and catechol

Ion Species	Log (*K*)[a]	Reference	Acidity (p*K*a)	Reference
Fe^{3+}	18.52	[6]	2.19	[7]
Al^{3+}	16.3	[8]	5	[9]
Cu^{2+}	13.59	[6, 10]	8.0	[7]

[a] K = {[ML]/[M][L]}, where M, L, and ML are metal ion, ligand, and their metal-ligand complex, respectively

ligand, and pKa value for metal ion acidity in aqueous state.

Supporting Reference

[S1] J. Yin, Z. Zhang, X. Li, J. Yu, J. Zhou, Y. Chen, W. Guo, *Nat. Commun.* **2014**, *5*, 3582.

[S2] J. Yin, X. Li, J. Yu, Z. Zhang, J. Zhou, W. Guo, *Nat. Nanotechnol.* **2014**, *9*, 378.

[S3] J. Park, S. Song, Y. Yang, S. H. Kwon, E. Sim, Y. S. Kim, *J. Am. Chem. Soc.* **2017**, *139*, 10968.

[S4] M. Z. Bazant, K. Thornton, A. Ajdari, *Phys. Rev. E - Stat. Physics, Plasmas, Fluids, Relat. Interdiscip. Top.* **2004**, *70*, 24.

[S5] R. Pierret, *Semiconductor Device Funtamentals*, Pearson Education, India **1996**.

[S6] M. J. Sever, J. J. Wilker, *Dalt. Trans.* **2006**, 813.

[S7] G. Galstyan, E. W. Knapp, *J. Comput. Chem.* **2015**, *36*, 69.

[S8] G. Dalla Torre, J. I. Mujika, E. Formoso, E. Matito, M. J. Ramos, X. Lopez, *Dalt. Trans.* **2018**, *47*, 9592.

[S9] E. Koubek, C. McWherter, G. L. Gilbert, *J. Chem. Educ.* **1998**, *75*, 60.

[S10] A. E. Martell, R. M. Smith, *Other Organic Ligands*, Springer Science+Business Media, New York, USA **1977**
